# Organization of Squamata (Reptilia) assemblages in Mediterranean archipelagos

**DOI:** 10.1002/ece3.6013

**Published:** 2020-01-22

**Authors:** Daniel Escoriza

**Affiliations:** ^1^ GRECO Institute of Aquatic Ecology University of Girona Girona Spain

**Keywords:** co‐occurrences, islet, lizard, phylogenetic dispersion, snake

## Abstract

Mediterranean islands have complex reptile assemblages, but little is known about the factors that determine their organization. In this study, the structure of assemblages of Squamata was evaluated based on their species richness and two measures of phylogenetic diversity (variability and clustering). I evaluated the composition of the assemblages comparing distinct biogeographic subregions within the Mediterranean: Adriatic, Aegean, Balearic, Corsica–Sardinia, Crete, Gulf of Gabés, Ionian Sea, Ligurian Sea, Malta, Sicily, and Tyrrhenian Sea. The effect of island environments and geographical isolation on the diversity metrics was assessed using generalized linear models. The analyses indicated that species richness was mostly influenced by island area and geographical isolation. Assemblages on smaller islands were poorer in species and phylogenetically dispersed, possibly as an effect of interspecific competition. The species composition of the assemblages was determined by similar environmental drivers within the biogeographic subregions, including island area, island elevation, geographical isolation, and aridity. In several subregions, significant patterns of phylogenetic attraction were found in species co‐occurrences, caused by the limits imposed by the island size on large predatory species.

## Graphical abstract

The effect of island environments and geographical isolation on the diversity metrics was assessed using generalized linear models. Assemblages on smaller islands were poorer in species and phylogenetically dispersed, possibly as an effect of interspecific competition. In several subregions, significant patterns of phylogenetic attraction were found in species co‐occurrences, caused by the limits imposed by the island size on large predatory species.

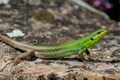

## 1 INTRODUCTION

During the late Neogene, the Mediterranean basin was subjected to cyclical sea level fluctuations caused by climatic instability (Peirano et al., 2004). Marine regressions facilitated the exchange of biota between islands and the continent, but throughout the Pleistocene some islands (e.g., the Balearic Islands) remained isolated (Bover, Quintana, & Alcover, 2008; Marra, 2013). Therefore, some Mediterranean islands support rich assemblages, composed by subsets of those from the nearest continental land, whereas others are poorer, predominantly composed by species arrived by Messinian dispersal (Sara & Morand, 2002). During the Holocene, enrichment and homogenization of the insular assemblages occurred following the beginning of the anthropic colonization of Mediterranean islands, which particularly affected bird and mammal faunas, but also large reptiles (Bonfiglio, Marra, & Masini, 2000; Vigne, 1992). The fauna of the Mediterranean islands was later affected by the maritime trade that followed expansion of the Roman Era at approximately 2,300 years before present (BP). This led to extensive translocation of species from the continent to the islands in a process that has continued to the present (Insacco, Spadola, Russotto, & Scaravelli, 2015; Spaneli & Lymberakis, 2014; Traveset et al., 2009).

The composition of biotic communities is determined by the island geographic isolation and colonization history but also by the constraints imposed by limited resources (Goldstein, 1975; MacArthur & Wilson, 1967). Insular ecosystems saturate faster than those on continents (Terborgh & Faaborg, 1980), and small islands typically contain impoverished assemblages composed of trophic generalists (Holt, Lawton, Polis, & Martinez, 1999). Island biotic communities are also characterized by their fragility and dynamism and can be rapidly unbalanced by the extinction or addition of a single species (Corlett, 2010; Simberloff, 2000).

**Figure 1:**
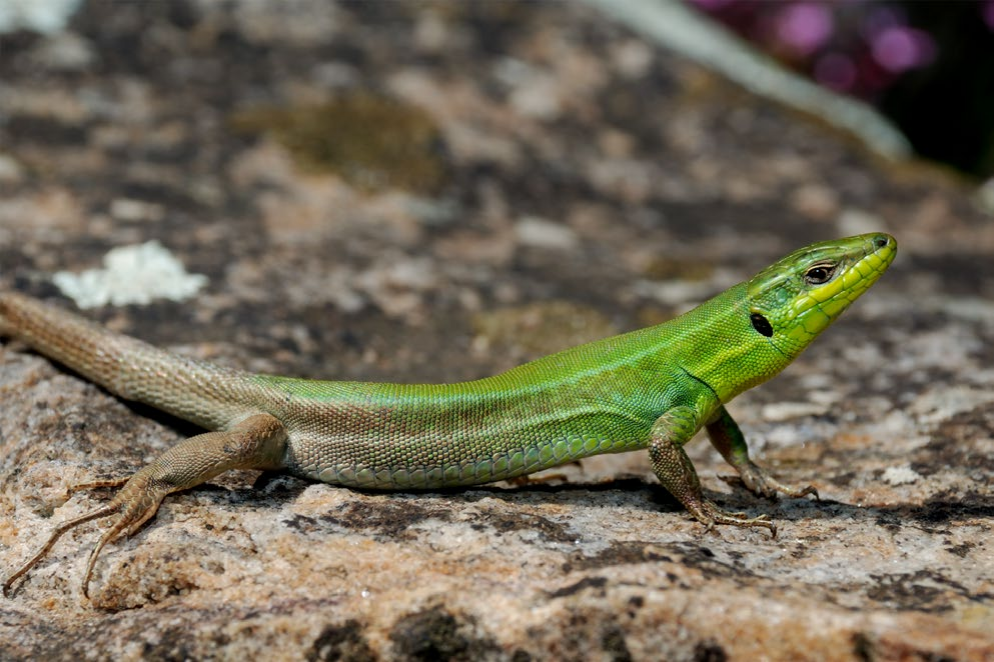
Organism photograph: The endemic island lizard *Podarcis waglerianus* (Cefalù, Sicily). Credit: Daniel Escoriza

Here, I studied insular assemblages of Squamata (Reptilia), focusing in the species diversity and the environmental effects on the assembly structure. Morphologically very divergent species were included in the study (i.e., “lizards", amphisbaenians, and snakes; Figure 1) because phylogenetic analyses demonstrated that the quadruped group typically referred to as “lizards” is paraphyletic (Reeder et al., 2015).

The patterns of species coexistence were used to test hypotheses on the effect of insularity. The effect on phylogenetic diversity was investigated to assess whether the assemblages were composed of close or distant relatives (Ives & Helmus, 2010). Phylogenetically dispersed assemblages are expected when competition drives species packing, as related species strongly overlap in the use of resources (Burns & Strauss, 2011; Morlon, Kefi, & Martinez, 2014). Phylogenetically clustered assemblages are expected if the traits that favor dispersal are shared among close relatives (Weigelt et al., 2015). In mammals, it has been shown that island assemblages are organized following a phylogenetic structure (Cardillo, Gittleman, & Purvis, 2008), but it is unknown if this also occurs with reptiles.

My hypothesis was that the Squamata assemblages would be poorer in species and phylogenetically structured on small isolated islands, because of habitat constraints and dispersal filtering (Hypothesis 1). On small and environmentally homogeneous islands, negative interspecific associations may also occur among species that are not phylogenetically close but are in the same trophic rank, or involve predators and their prey (Savidge, 1987). Such interactions have been invoked to explain the assemblage composition on several Mediterranean islands (Grano, Cattaneo, & Cattaneo, 2013; Pérez‐Mellado, Corti, & Lo Cascioa, 1997). For this reason, species co‐occurrences determined by phylogenetic relationships and negative interspecific associations were expected to appear (Hypothesis 2).

## 2 MATERIAL AND METHODS

### 2.1 Study system

**Figure 2:**
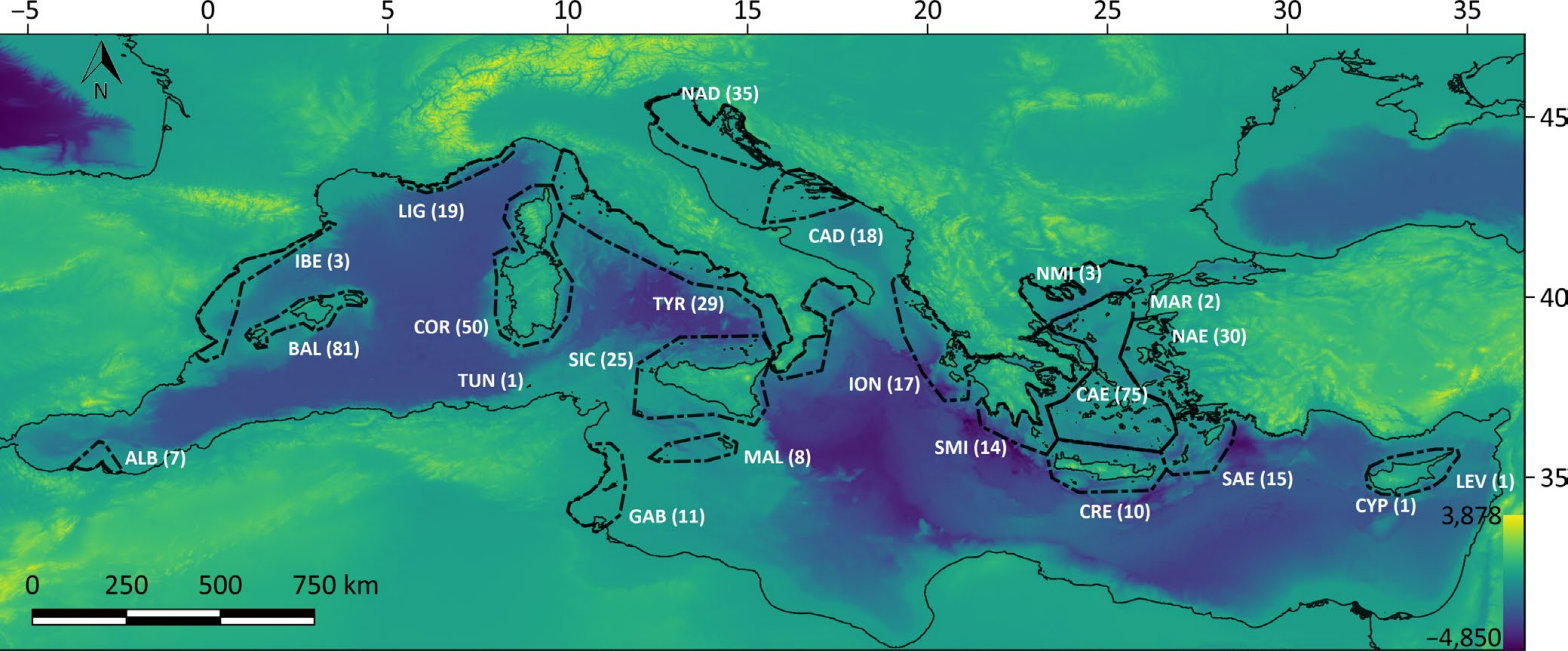
Map of the study region showing the biogeographical subregions and the respective number of islands (in brackets). ALB, Alboran sea; BAL, Balearic sea; CAD, central Adriatic; CAE, central Aegean; COR, Corso‐Sardinian; CRE, Crete; CYP, Cyprus; GAB, Gulf of Gabès; IBE, mainland Spain; ION, Ionian sea; LEV, Levantine sea; LIG, Ligurian‐Provence; MAL, Malta‐Lampedusa; MAR, Marmara sea; NAD, northern Adriatic; NAE, northern Aegean; NMI, northern mainland Greece; SAE, southern Aegean; SIC, Sicily‐Aeolian; SMI, southern mainland Greece; TUN, Tunisia; TYR, Tyrrhenian sea (mainland Italy)

The study region is the basin of the Mediterranean Sea (2.5 × 10^6^ km^2^). This region includes islands ranging in size from 1,000 m^2^ (Torre Scuola, Liguria) to 25,711 km^2^ (Sicily). Mediterranean islands have a high diversity of Squamata that exceeds 20 species in those of the Aegean and Ionian seas (Chondropoulos, 1986, 1989). The predominant climate in the region is Mediterranean, involving two Köppen classification subtypes (*Csa*, and the transitional temperate variant *Csb*), although in the southernmost islands (e.g., Alboran, Formentera, Pantelleria, Lampedusa, Cyprus, and Salamis) the climate is classified as steppic (Kriticos et al., 2012). The biotic diversity of the Mediterranean basin is structured in well‐defined biogeographical subregions (Coll et al., 2010; Kougioumoutzis et al., 2017; Lloret et al., 2005). In this study, the subregion borders proposed in these studies were used (Figure 2).

### 2.2 Assemblage composition and phylogenetic data

Data on the occurrence of 104 species of Squamata on 455 islands were obtained from biogeographic atlases and scientific papers (see the references provided in Supporting Information 1). Taxonomic classification followed Speybroeck, Beukema, Bok, and Voort (2016) and Uetz, Freed, and Hošek (2019). The phylogenetic relationships between species pairs were obtained using the TimeTree database (Kumar, Stecher, Suleski, & Hedges, 2017). This database provides estimates of the times of divergence between species pairs, based on a synthesis of previous studies (Hedges, Marin, Suleski, Paymer, & Kumar, 2015). The phylogenetic tree built using MEGA‐X 10.0.5 (Kumar, Stecher, Li, Knyaz, & Tamura, 2018) is shown in Supporting Information 2.

The matrix of pairwise phylogenetic distances was used to calculate the phylogenetic species variability (PSV) and phylogenetic species clustering (PSC) (Helmus, Bland, Williams, & Ives, 2007). PSV is statistically independent of species richness and measures the mean relatedness among all the species composing an assemblage (Helmus et al., 2007). PSC measures the phylogenetic distance between the nearest relatives within an assemblage. Both metrics tend to a value approaching zero when the species are phylogenetically close and to a value of one if they are not closely related (Helmus et al., 2007). These metrics were calculated using the picante package (Kembel et al., 2010) in the R environment (R Core Development Team, 2019).

### 2.3 Environmental data

Several ensembles of environmental variables were selected because of their likely influence on island biotic communities and reptile occurrence in the Mediterranean ecoregion (Escoriza, 2018; Raposeiro, Hughes, & Costa, 2013). These variables describe the physical (surface area and elevation) and climate characteristics of the islands, and their geographical isolation relative to the continent or larger islands. The physical data for the islands were obtained based on atlases (Koster, 2005) and Google Earth Pro 7.3.2.5776 (Google LLC). Climate was characterized using an aridity index (the ratio between annual accumulated precipitation and potential evapotranspiration, with higher values indicating a lower deficit of environmental water) and mean annual temperatures (measured in °C), both obtained from GIS‐modelled data (Fick & Hijmans, 2017; Zomer, Trabucco, Bossio, & Verchot, 2008). The data (ESRI Grid, 1,000 m pixel^−1^ resolution) were downloaded from the WorldClim Version2 database (https://worldclim.org/version2) and Global Aridity and PET database  (https://cgiarcsi.community/data/global-aridity-and-pet-database/).

The geographical isolation of each island was characterized based on the shortest distance (in km) of the island from the mainland and larger islands (i.e., those larger than 5 km^2^) and the average depth of the sea (in m) within a 5–30 km radius of the island. The geographical distances were measured using Google Earth Pro. The mean depth of the seabed is a proxy for the susceptibility of these islands to be colonized during regression of the epicontinental sea (Chiocci, Ercilla, & Torre, 1997). The mean depth of the sea was calculated from a digital model of the sea floor (Becker et al., 2009), downloaded from the GEBCO database (https://www.gebco.net/). Data from GIS databases were extracted using the Quantum‐GIS 3.6.0 package (QGIS Development Team, 2019).

### 2.4 Data analysis

The analyses evaluated: (a) the effect of the island environment and its geographical isolation on the diversity and species composition of the assemblages, and (b) patterns of species spatial associations (co‐occurrences), and the phylogenetic structure. Prior to analysis, highly correlated predictors (*r* ≥ 0.75) were removed following construction of a correlation matrix. Variables that showed absolute skew and kurtosis values indicating non‐normality were logarithmically transformed, if data were highly skewed to the right (Lewis, 1977). All predictor variables were normalized.

The association between the environmental predictors and diversity metrics (species richness, PSV, and PSC) were evaluated using generalized linear models (GLMs). The relative contribution of the pooled variables was evaluated using automated model selection and model averaging for GLMs (Calcagno & de Mazancourt, 2010). The best candidate models were obtained using the set of all variables, and the models were ranked using the small‐sample‐corrected Akaike information criterion (AICc; Burnham & Anderson, 2002). The statistical importance of the variables was determined according to their model‐averaged weighting in the 100 best models. These analyses were conducted using the glmulti (Calcagno & de Mazancourt, 2010) package in R.

The effect of the environmental gradient on the composition of an assemblage (species presence/absence matrix) was evaluated between those sets of species that overlap geographically, grouped by subregions. This analysis was conducted using distance‐based redundancy analysis (dbRDA) (Legendre & Anderson, 1999), after transformation of the binary matrix of species occurrences into a matrix of Sørensen distances (Faith, Minchin, & Belbin, 1987). Determination of the most significant associations was based on the AIC‐like statistics and forward variable selection after 999 permutations (Legendre, Oksanen, & Braak, 2011). These analyses were conducted using the vegan package (Oksanen et al., 2019) in R.

The association between co‐occurrences and phylogenetic distances was evaluated within the biogeographic subregions that comprised at least eight islands. The interspecific distances based on co‐occurrences were obtained using the Schoener *Cij* index (Schoener, 1970), and the effect of phylogenetic distance was determined using quantile regression (Lovette & Hochachka, 2006). This effect was assessed for the 25, 50, and 75th percentiles of the *Cij* distribution. The statistical significance was determined using a null model that maintained sample species richness (Savage & Cavender‐Bares, 2012). The statistical significance of the co‐occurrence of species was determined using probabilistic analysis (Veech, 2013). These analyses were conducted using the picante and co‐occur packages (Griffith, Veech, & Marsh, 2016) in R.

## 3 RESULTS

The correlation matrix showed that no variable had a very high correlation (*r* ≥ 0.75) with those previously included in the models, so no variable was removed from the analysis. The results of the GLM analysis showed that the variables best explaining the variation in species richness were island area and mean annual temperature, which had positive influences, and mean sea depth in a 5 km radius and the distance to the continent or larger islands, which had negative influences (Table 1). The variables best explaining variation in the PSV were the aridity index and island area (negative influences), and mean sea depth in a 5–30 km radius and distance to the continent (positive influences; Table 1). The variables best explaining variation in the PSC were island area and the aridity index (negative influences), and mean sea depth in a 5 km radius and distance to the continent or larger islands (positive influences; Table 1).

**Table 1:** General patterns of diversity of Squamata in the Mediterranean islands, assessed by automated GLM selection

Diversity metric	Best subset of predictors	*z*‐value	*p*	IMP_100_
Species richness	Island area	27.13	2^−16^	1.00
	Distance to larger island	–3.89	.0001	0.99
	Distance to continent	–2.74	.006	0.92
	Mean sea depth 5 km	–2.47	.014	0.80
	Mean temperature	2.08	.038	0.61
Phylogenetic species variability	Aridity index	–6.08	3^−9^	1.00
	Island area	–5.16	4^−6^	0.99
	Distance to continent	4.02	.00007	0.99
	Mean sea depth 30 km	1.89	.06	0.77
	Mean sea depth 5 km	1.64	.102	0.65
Phylogenetic species clustering	Island area	–15.13	2^−16^	1.00
	Distance to continent	3.64	.0003	0.99
	Mean sea depth 5 km	3.62	.0003	0.98
	Aridity index	–3.97	.00009	0.96
	Distance to larger island	2.55	.01	0.90

Note

The statistics of the best candidate model and the model‐averaged importance (IMP_100_) for the variables in the 100 best models are shown.

**Figure 3:**
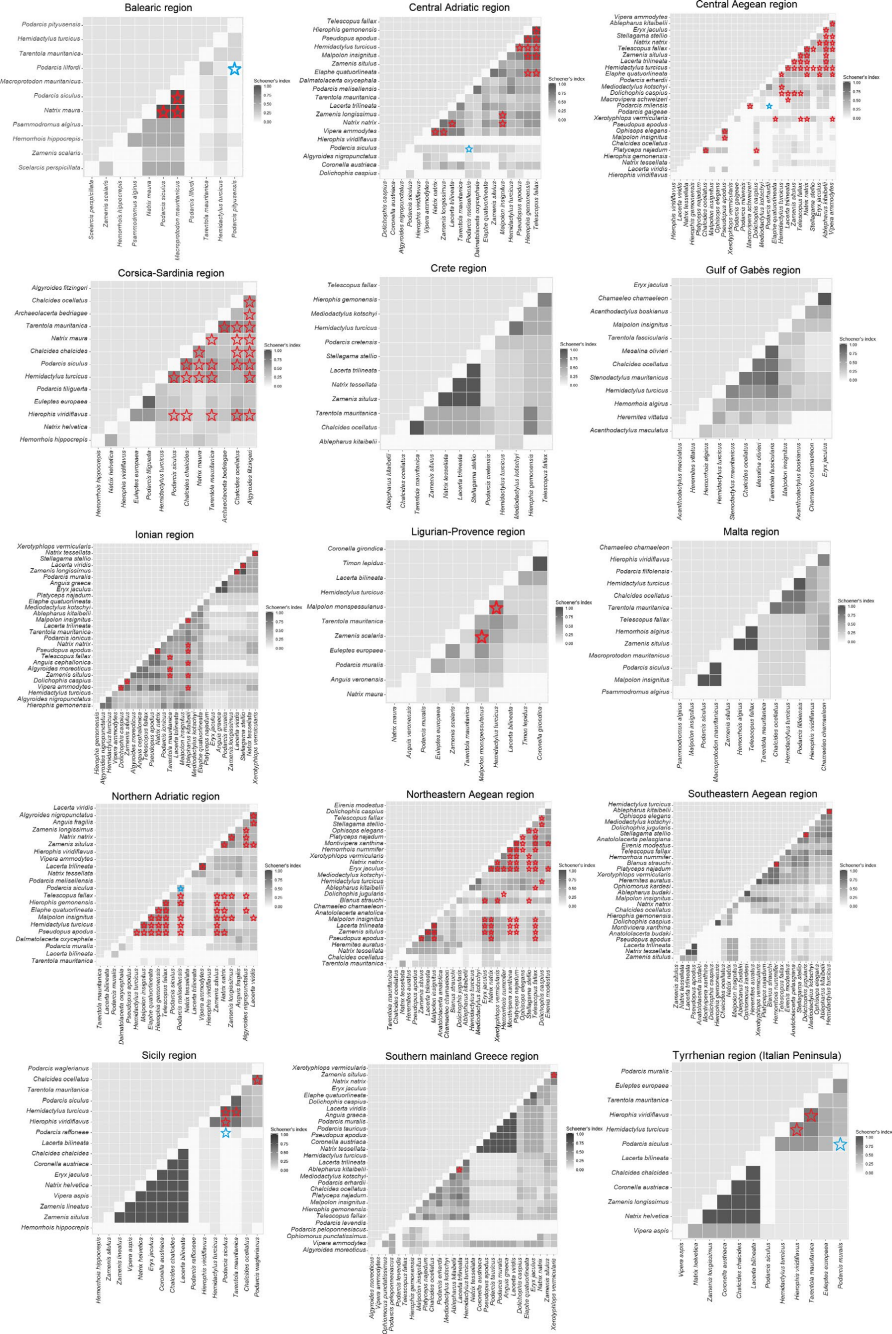
Co‐occurrence matrices, based on the Schoener's Index, generated for each subregion with more than eight islands. The red star indicated the statistically significant positive associations and the blue star the statistically significant negative associations (*α* = 0.01) according to a probabilistic analysis

The species composition of the assemblages within subregions was largely determined by the island area (91.7% of subregions), aridity index (33.3%), mean sea depth in a 30 km radius (25%), mean sea depth in a 5 km radius (16.7%), island elevation (16.7%), distance to the continent (8.3%), and mean annual temperature (8.3%) (Table 2). Quantile regression indicated that there was a random phylogenetic effect on the co‐occurrences, with several exceptions in the upper quartile (Table 3). All significant effects involved a negative association between co‐occurrence and phylogenetic distance (phylogenetic attraction) (Table 3). Most of the co‐occurrences showed no statistical significance (Figure 3). Significant positive associations were detected between several pairs of species of snake and lizard (Figure 3). Significant negative associations were only detected between some pairs of congeneric lizards (genus *Podarcis*; Figure 3 and Table 4).

**Table 2:** Results of the distance‐based redundancy analysis assessing the effect of island characteristics and the assemblage composition within subregions

Subregion	Predictors	*R* ^2^ adjusted	AIC	*F*	*p*
Balearic	Distance to continent	0.62	175.9	133.8	.002
	Island area	0.67	166.9	11.3	.004
	Mean sea depth 30 km	0.68	164.6	4.3	.044
Central Adriatic	Island area	0.30	21.0	8.1	.002
	Aridity index	0.40	19.0	3.7	.01
Central Aegean	Island area	0.17	193.5	16.5	.002
	Aridity index	0.23	189.1	6.4	.004
	Mean sea depth 30 km	0.26	187.1	3.9	.002
	Mean sea depth 5 km	0.29	185.7	3.3	.022
Corsica‐Sardinia	Island area	0.40	61.7	33.2	.002
	Annual temperature	0.44	58.7	4.9	.022
Ionian Sea	Mean sea depth 30 km	0.14	24.6	3.6	.006
	Aridity index	0.29	22.1	4.2	.004
	Island area	0.47	18.0	5.6	.002
Ligurian Sea‐Provence	Island area	0.18	12.7	5.0	.008
	Aridity index	0.33	9.7	4.9	.006
Northern Adriatic	Island elevation	0.29	61.0	15.2	.002
Northeastern Aegean	Island area	0.28	51.3	12.2	.002
	Island elevation	0.32	50.4	2.8	.046
Southeastern Aegean	Island area	0.27	14.8	6.1	.002
Sicily	Island area	0.17	28.6	5.9	.002
	Mean sea depth 5 km	0.36	23.0	7.9	.002
South mainland Greece	Island area	0.11	15.7	2.5	.03
Tyrrhenian Sea (Italian Peninsula)	Island area	0.18	45.4	7.2	.004

Note

Some subregions are not shown because no statistically significant associations were found at *α* = 0.05.

**Table 3:** Association between phylogenetic distance and the co‐occurrence metric *Cij*, evaluated by quantile regression

Subregion		*Q*_25_	*Q*_50_	*Q*_75_
Balearic	Slope	0.0001	0.0002	−0.0006
	*p*	0.88	0.97	0.001
Central Adriatic	Slope	0.0006	0.0001	−0.0003
	*p*	0.999	0.787	0.074
Central Aegean	Slope	0.00	0.00007	0.0001
	*p*	0.605	0.871	0.907
Corsica‐Sardinia	Slope	−0.0001	0.0004	0.0003
	*p*	0.294	1.00	0.997
Crete	Slope	0.00	−0.0002	−0.0005
	*p*	0.380	0.164	0.066
Gulf of Gabès	Slope	0.00006	−0.0002	0.0005
	*p*	0.751	0.255	0.954
Ionian Sea	Slope	−0.0002	−0.0001	0.00
	*p*	0.198	0.162	0.512
Ligurian Sea‐Provence	Slope	0.000001	0.0001	0.00004
	*p*	0.483	0.721	0.665
Malta	Slope	0.001	0.001	−0.001
	*p*	0.781	0.968	0.001
Northern Adriatic	Slope	−0.0002	−0.0002	−0.0004
	*p*	0.168	0.132	0.015
Northeastern Aegean	Slope	−0.0005	−0.0008	−0.0007
	*p*	0.007	0.001	0.001
Southeastern Aegean	Slope	0.0004	0.0001	0.00
	*p*	0.997	0.763	0.495
Sicily	Slope	0.0001	0.00	−0.002
	*p*	0.889	0.468	0.001
South mainland Greece	Slope	−0.0001	−0.0003	0.00
	*p*	0.184	0.018	0.469
Tyrrhenian Sea (Italian Peninsula)	Slope	0.00004	−0.002	−0.001
	*p*	0.683	0.001	0.001

Note

The slope values and their statistical significance are shown for each of the measured quantiles (*Q*) of the distribution of *Cij*.

**Table 4:** Species pairs of significant negative associations, as determined by probabilistic co‐occurrence analysis

Subregion	Species 1	Species 2	AI	EI	*p*
Balearic	*Podarcis lilfordi*	*Podarcis pityusensis*	0	16.0	.00001
Central Adriatic	*Podarcis siculus*	*Podarcis melisellensis*	1	4.3	.0007
Central Aegean	*Podarcis erhardii*	*Podarcis milensis*	0	5.2	.0001
Northern Adriatic	*Podarcis siculus*	*Podarcis melisellensis*	12	14.8	.01
Sicily	*Podarcis siculus*	*Podarcis raffoneae*	1	3.4	.0067
Tyrrhenian Sea (Italian Peninsula)	*Podarcis siculus*	*Podarcis muralis*	2	7.6	.00001

Abbreviations: AI, actual number of islands with both species; EI, expected number of islands with both species, if the two species occurred randomly.

## 4 DISCUSSION

The Mediterranean archipelagos provide an interesting example of the interacting mechanisms that shape island biotic communities, for their physical heterogeneity and distinct colonization histories (Blondel, Chessel, & Frochot, 1988). In this study, the analyses showed that the area of the islands and their geographical isolation determined the diversity of the Squamata assemblages, as expected. These factors were of similar importance and, for this reason, large and orographically heterogeneous islands (e.g., Crete, Mallorca, Sardinia, and Sicily) show relatively species‐poor assemblages compared with Ionian or Aegean islands (e.g., Korfú or Samos), which are smaller but situated close to the continent. Crete, Mallorca, and Sardinia were completely isolated by the end of the Messinian age and had impoverished and unbalanced faunas until the Late Pleistocene—Holocene (Krijgsman, Hilgen, Raffi, Sierro, & Wilson, 1999; Melis, Palombo, Ghaleb, & Meloni, 2016; Meulenkamp, Wortel, Wamel, Spakman, & Strating, 1988). Although currently situated very close to the continent, Sicily possibly had its colonization hindered by the fragmentation of Calabria into several islands until the mid‐Pleistocene, and the continuous persistence of the Strait of Messina throughout the Late Glacial (Marra, 2009; Palombo, 2018).

The colonization history has left its footprint in the organization of the Squamata assemblages. The analyses indicated that islands at a greater distance from the continent and surrounded by deeper water have comparatively species‐poorer and phylogenetically more dispersed assemblages, according to both PSV and PSC. This suggests that in the Mediterranean no Squamata lineages are having greater dispersal/colonizing capacity than others. Islets also showed impoverished assemblages, mostly composed of distantly related species. Many of these islets are situated near the continent, so could be colonized during the glacial‐eustatic marine regressions by mainland species (Fattorini, 2010; Kougioumoutzis et al., 2017). The simplified reptile assemblages on these islets could be the result of interspecific competition, intensified after the separation of the islet from the continental land. Similarly, higher levels of aridity were associated with phylogenetic dispersed assemblages. This could be the consequence of more intense competitive interactions caused by the lower primary productivity in dry environments (Le Houérou, Bingham, & Skerbek, 1988), but could also reflect the presence of more distinct evolutionary lineages in the southern Mediterranean region (Pyron, Burbrink, & Wiens, 2013).

Analysis of the effect of the environmental gradient on species occurrences supported these general conclusions. The area and topography of the islands, their relative geographical isolation, and the aridity determined the species identity in the assemblages in the distinct Mediterranean subregions. This is because these factors determined the dispersal success and the long‐term persistence of island populations, following the disappearance of land bridges (Foufopoulos, Kilpatrick, & Ives, 2010).

Co‐occurrence analysis revealed phylogenetic attraction, mostly in the upper quartile, for the Balearic, Sicilian, north Adriatic, northern Aegean, and Maltese archipelagos, and the Tyrrhenian pericontinental islands. The larger islands of these archipelagos included several cohorts of species that are completely absent on islands that are about 5 km^2^ or less in size (Chondropoulos, 1989; Fattorini, 2010; Koren, Laus, Buric, & Kuljeric, 2011; Lanfranko, 1955; Massa, 2008; Pinya & Carretero, 2011; Tóth, Grillitsch, Farkas, Gál, & Sušić, 2006). These cohorts are mostly composed of colubrid snakes occupying higher trophic ranks and/or with specialized diets (Geniez, 2015). The effect of the species area of the islands could be greater for predatory species and those having restricted diets, and consequently several species of snake could not maintain populations on small islands (Holt et al., 1999). This has been empirically demonstrated for island populations of some Mediterranean snakes, and these species adapt by shifting their diet or reducing body size (Filippi, Capula, & Luiselli, 2003; Luiselli, Petrozzi, Mebert, Zuffi, & Amori, 2015).

Co‐occurrence analyses also indicated that predator—prey interactions do not impose major restrictions on island coexistence, at least on medium to large islands. These analyses revealed examples where predators and prey positively co‐occurred throughout the entire island network (e.g., *Podarcis milensis*—*Macrovipera schweizeri*; Adamopoulou, Valakos, & Legakis, 1997). Some of the prey species have developed mechanisms for predation avoidance (e.g., tail autotomy) that reduce the demographic impact of predators (Pafilis, Foufopoulos, Poulakakis, Lymberakis, & Valakos, 2009). Snakes and their prey also coexist on some satellite islets, but the snake populations are fragile and prone to extinction if the availability of prey decreases (e.g., associated with campaigns to control rat populations; Slavenko, Tallowin, Itescu, Raia, & Meiri, 2016; Vanni & Nistri, 2006).

The analysis revealed only significant negative associations between some species of the *Podarcis* genus. These species commonly occupy very small islets (Brown & Pérez‐Mellado, 1994; Raia et al., 2010), where the extremely simplified habitats do not support the coexistence of ecologically homologues species. Some of these congeneric lizards can coexist on large islands, but they segregate parapatrically among macrohabitats (Delaugerre & Cheylan, 1992; Tóth et al., 2006); when they co‐occur on smaller islands the result is rapid extinction of one of the species (Nikolic et al., 2019).

This study has provided new insights into the organization of Squamata assemblages on Mediterranean islands. These islands include numerous endemic species, particularly of small lacertidae of the genus *Podarcis* (Itescu et al., 2018). The negative association patterns found in the co‐occurrence analysis suggest that the introduction of alien congeneric species could have a very negative effect on microinsular endemic lizards. Accidental translocation of several species of *Podarcis* has been documented in the Mediterranean (Silva‐Rocha et al., 2014; Spilani et al., 2018). The results of this study indicate that in the event of the introduction of alien lizards to small islets, their rapid control and eradication is advisable.

## 5 CONCLUSION

Phylogenetic relationships have an important effect on the organization of biotic communities. The Squamata assemblages in small islands are phylogenetically evenly dispersed. Pairs of congeneric species are associated negatively in small island systems; therefore, accidental translocations of mainland related species can have a pernicious effect on island endemisms. The results of this study, in addition to disentangling the relationships between assemblage organization and phylogenetic diversity, have practical value for conservation management of island reptile faunas.

## CONFLICT OF INTEREST

None declared.

## AUTHOR CONTRIBUTION

Daniel Escoriza conceived and wrote the manuscript.

## Data Availability

Dataset for island environmental characteristics and composition of reptile assemblages available at Dryad Digital Repository: https://doi.org/10.5061/dryad.9ghx3ffdc.
