# Supplementary figures and images for: Organization of Squamata (Reptilia) assemblages in Mediterranean archipelagos
Source: Ecol Evol. 2020 Jan 22;10(3):1592–601. doi: 10.1002/ece3.6013 (PMC7029066; doi:10.1002/ece3.6013)

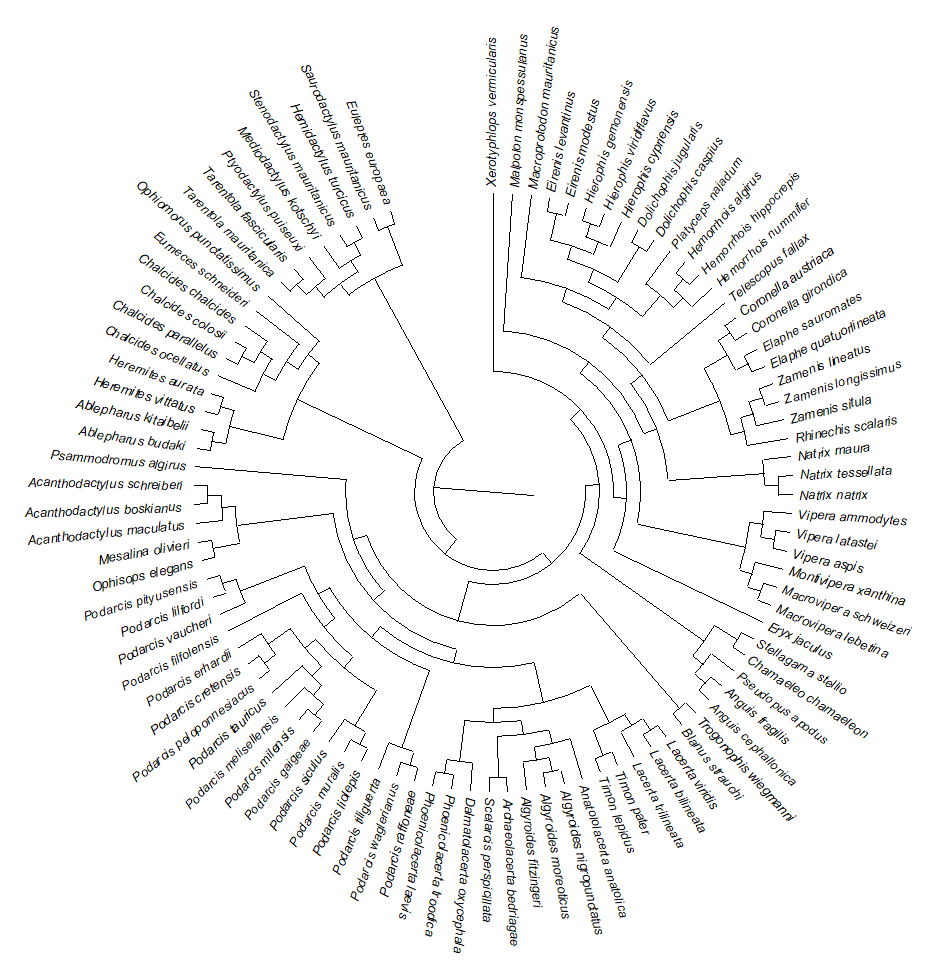

Supplement: Supplementary file 2 [file ECE3-10-1592-s002.tif]
